# Three-dimensional laser combined with C-arm computed tomography-assisted puncture of intracerebral hemorrhage

**DOI:** 10.3389/fendo.2023.1198564

**Published:** 2023-06-28

**Authors:** Hongwei Zhao, Tao Zhang, Min Li, Yang Gao, Shuangquan Wang, Rongcai Jiang, Zefu Li

**Affiliations:** ^1^ Tianjin Medical University, Tianjin, China; ^2^ Department of Neurosurgery, Binzhou Medical University Hospital, Binzhou, China; ^3^ Department of Neurosurgery, Tianjin Medical University General Hospital, Tianjin, China; ^4^ Department of Ultrasound Medicine, Binzhou Medical University Hospital, Binzhou, China; ^5^ Tianjin Neurological Institute, Key Laboratory of Post-Neuroinjury Neuro-repair and Regeneration in Central Nervous System, Tianjin Medical University General Hospital, Ministry of Education, Tianjin, China

**Keywords:** hypertensive intracerebral hemorrhage, C-arm CT, three-dimensional laser, hematoma puncture drainage, minimally invasive technology

## Abstract

**Background:**

Intracerebral hemorrhage (ICH) is the deadliest subtype of stroke, with a 30-day case fatality rate of approximately 40%. Timely and accurate treatment is essential to facilitate recovery. The introduction of stereotactic instruments and navigation systems has greatly improved the accuracy of surgical treatment. In this study, we explored the application and effects of a three-dimensional (3D) laser combined with C-arm computed tomography (CT) on ICH puncture.

**Materials and methods:**

According to the principle of randomness, 118 patients with ICH were divided into control and experimental groups. The control group was treated with CT-guided puncture, and the experimental group was treated with 3D laser combined with C-arm CT puncture. The hematoma clearance rates at 3, 5, and 7 days after surgery and the prognosis at 1, 3, and 6 months after surgery were compared between the two groups.

**Results:**

The hematoma clearance rates of the group using 3D laser combined with C-arm CT at 3, 5, and 7 days after surgery were significantly higher than those of the control group, and the difference was statistically significant (*p* < 0.05). One month postoperatively, the daily living ability (ADL) grading and recovery of the patients in the test group was significantly better than those of the control group (*p* < 0.05), but there was no statistically significant difference in ADL 3 and 6 months after surgery (*p* > 0.05).

**Conclusion:**

3D laser combined with C-arm CT puncture has the advantages of real-time guidance, accurate positioning, and simple operation. It is an effective minimally invasive surgical method that is easy to master.

## 1 Introduction

Intracerebral hemorrhage (ICH) accounts for approximately 10%–20% of all strokes and is associated with greater morbidity and mortality compared to that of ischemic strokes (1). Hypertensive arteriopathy (deep perforator arteriopathy) and cerebral amyloid angiopathy are the two major risk factors of spontaneous ICH (2). Rapid identification and treatment are essential to facilitate recovery (3). Most patients who survive ICH have disabilities and are at risk of cognitive decline, recurrent stroke, and even systemic vascular disease. To date, no specific interventions have been shown to improve clinical outcomes after ICH (4). ICH is a great burden for patients, caregivers, family members, and society (5, 6). Surgical hematoma drainage has many theoretical advantages, such as prevention of cerebral herniation, reduction in intracranial pressure, and a decrease in excitotoxicity and neurotoxicity of blood products. Currently, the common surgical methods for ICH include craniotomy, decompressive craniectomy, neuroendoscopy, and minimally invasive catheter drainage after thrombolysis (7). Increasing evidence suggests that with minimally invasive procedures, the potentially adverse effects of open surgery in patients with ICH can be avoided, and a beneficial effect on the functional outcome may be achieved (8). We adopted a three-dimensional (3D) laser combined with C-arm computed tomography (CT) technology to puncture ICH, and compared it with the traditional CT-guided puncture of ICH to explore the reliability and effectiveness of 3D laser combined with C-arm CT puncture technology for minimally invasive puncture and drainage of hypertensive ICH.

## 2 Materials and methods

### 2.1 Patient selection

Data from 118 individuals with ICH treated at our center from January 2018 to June 2020 were analyzed retrospectively. The inclusion criteria were as follows: (A) all patients were definitely diagnosed with the first cerebral hemorrhage by brain CT, (B) patients had 30–60 ml basal ganglia hemorrhage without cerebral hernia, and (C) hematoma site was relatively concentrated. The exclusion criteria consisted of the following: (A) unstable blood pressure and heart rate, (B) severe underlying diseases, or (C) coagulation dysfunction. There were 59 men and 59 women, aged 40–88 years, with an average age (± standard deviation) of 61.25 ± 10.94 years. According to the random number table, the patients were divided into a control group and a test group, with 59 patients in each group. There were 27 men and 32 women in the control group, aged 42–86 years, with an average age of 61.54 ± 10.96 years. The experimental/test group consisted of 32 men and 27 women, aged 40–88 years, with an average age of 60.95 ± 11.01 years. The control group underwent hematoma puncture under CT guidance. The hematoma in the experimental group was punctured using 3D laser and C-arm CT. On statistical analysis, the general data of the two groups showed no significant differences (*p* > 0.05). The procedure was reviewed and approved by the Ethics Committee of the Binzhou Medical University Hospital. Consent for the operations was obtained from each patient and patient’s family.

### 2.2 Experimental instrument

**Figure 1:**
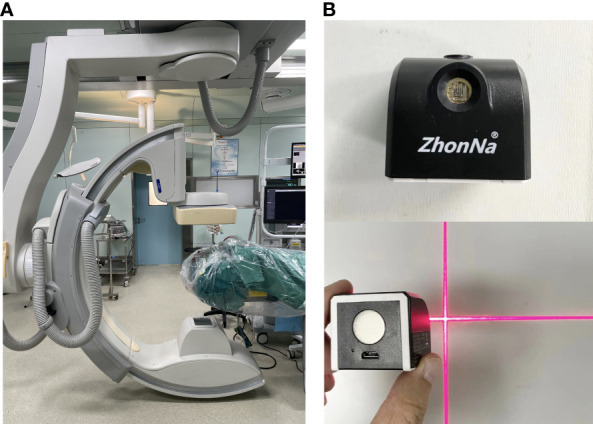
**(A)** Xper CT equipment. **(B)** Laser locator.

Instrumentation employed included Xper CT equipment (UNIQ FD20, made by Philips Medical Systems Nederland B.V.) and Laser locator (ZhonNa NX-9575-675, made by ZhonNa Electronics Company of Zhongshan) (**Figure 1**).

### 2.3 The control group underwent hematoma puncture under CT guidance

Points were marked on the frontal and temporal parts of the head of patients, and brain CT was used to ensure that the two marked points were located on the largest section of the hematoma. The connecting line between the two marked points was designated as Line A. The line between the hematoma center and the forehead marking point was defined as Line B, for which the length was measured, and the angle between line B and the center line was α. When puncturing, the forehead mark point was set as the puncture point, the drainage tube was punctured with a needle core along the direction of line A, and the angle between the drainage tube and midline was α. Simultaneously, we ensured that the depth of the drainage tube was the length of line B.

### 2.4 The experimental group underwent hematoma puncture under the guidance of 3D laser navigation and C-arm CT

#### 2.4.1 Software operation

**Figure 2:**
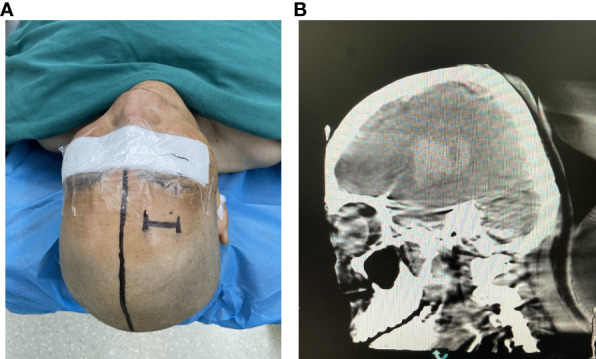
**(A)** The percutaneous puncture point was marked. **(B)** Confirming the puncture point in the CT image.

**Figure 3:**
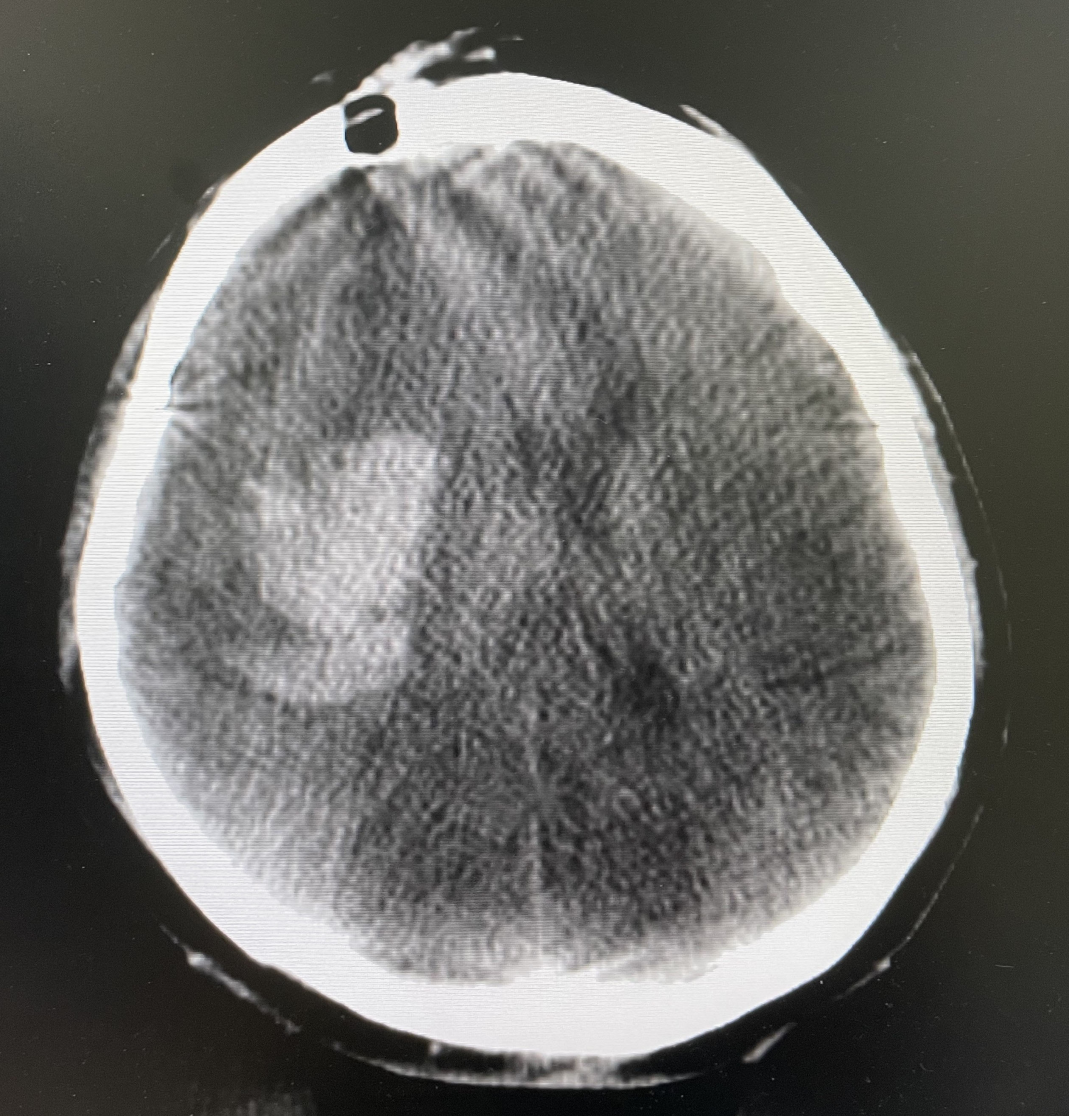
Skull burr hole.

**Figure 4:**
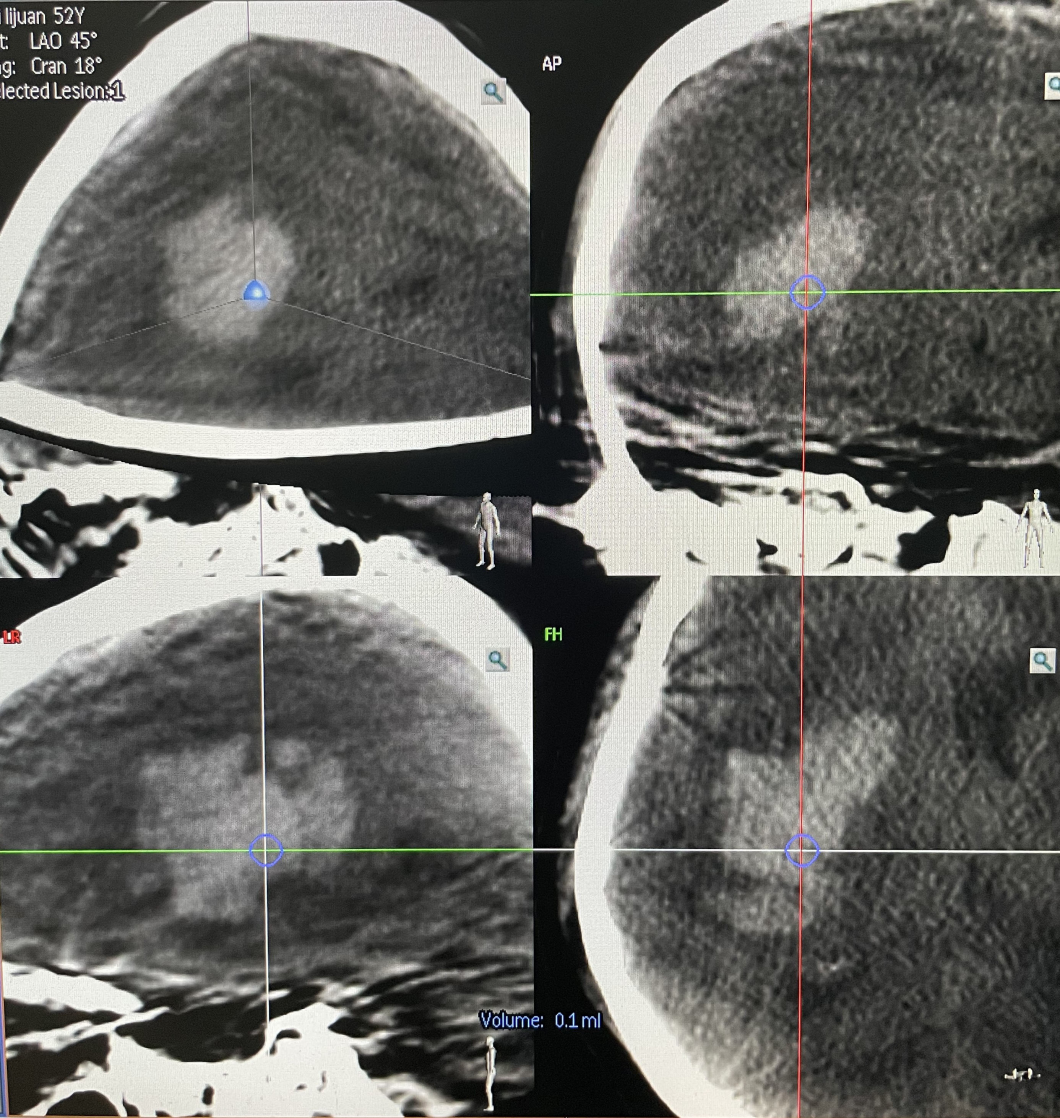
Setting the puncture.

**Figure 5:**
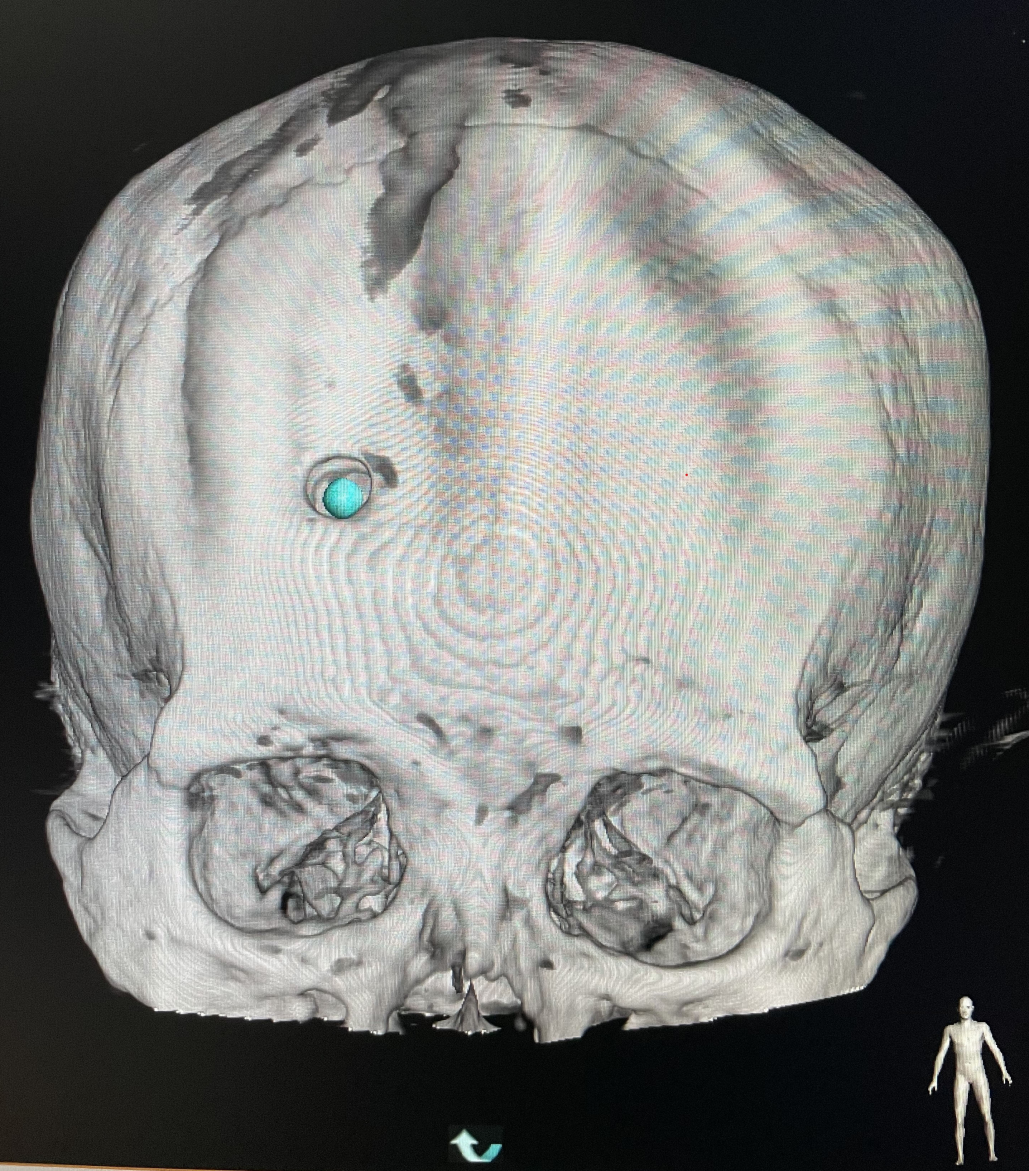
Overlapping the puncture point and puncture target.

**Figure 6:**
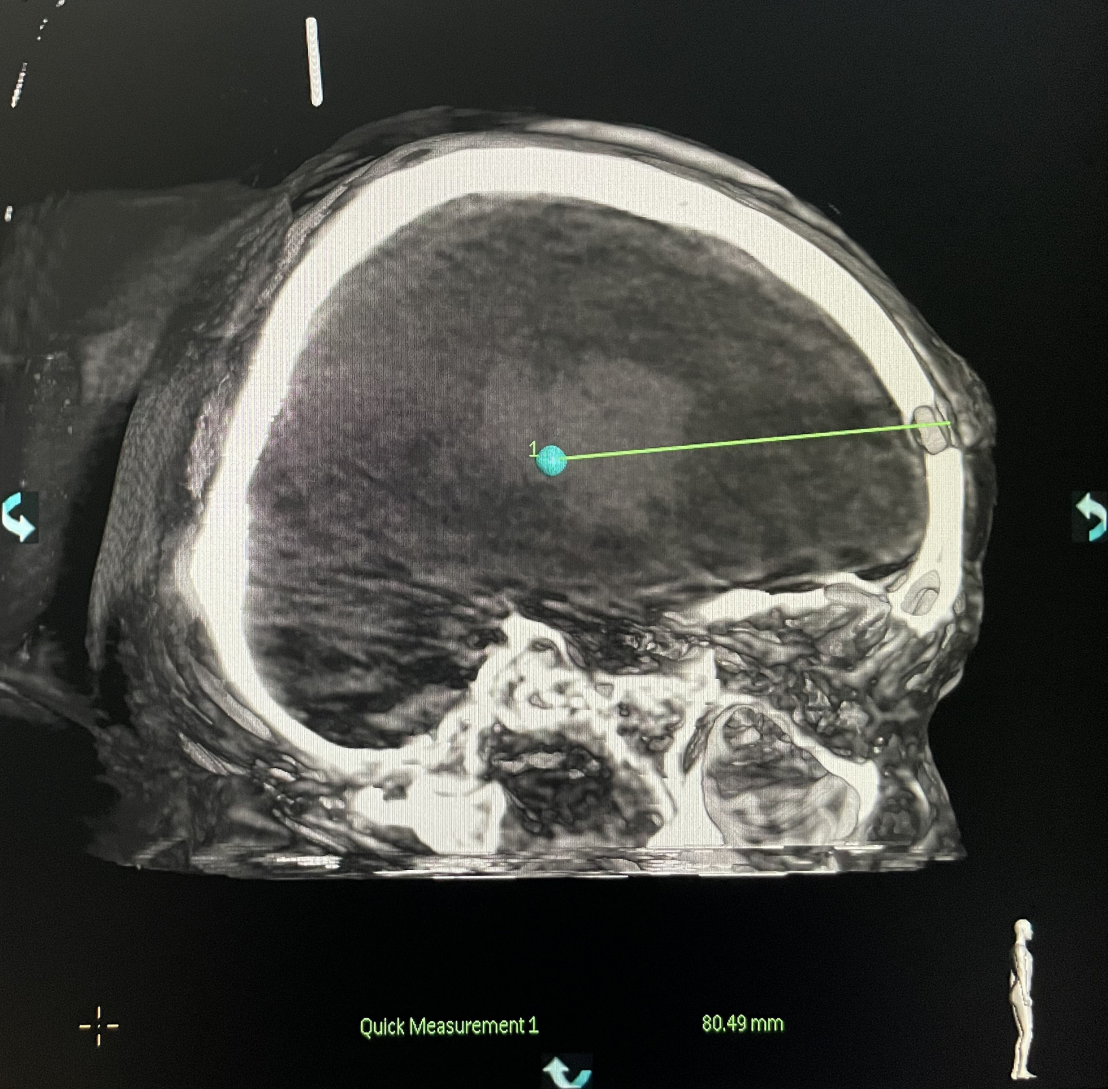
Measurement of the puncture depth.

The surgical incision site was marked with a metal object on the forehead, approximately 2 cm from the midline and 3 cm from the orbit on the hematoma side (**Figure 2**). The priority was to make an incision in the frontal stria and avoid the frontal sinus to prevent cerebrospinal fluid leakage after surgery and ensure an aesthetically pleasing appearance after wound healing. A hole was drilled in the bone at the metal mark (**Figure 3**). The C-arm CT function of the digital subtraction angiography (DSA) machine was used to collect the original data for processing; display the coronal, sagittal, and axial CT images; mark the center of the hematoma; and set it as the target site for the puncture (**Figure 4**). The 3D reconstruction of brain tissue was performed using the 3D reconstruction software Xper CT of the DSA machine, and the bone hole displayed in gray scale was adjusted as the puncture point. Subsequently, the 3D stereo image was rotated, overlapping the puncture point and puncture target. The laser emission direction was determined using the principle of “two points and one line,” and the real-time 3D reference image working angle was recorded (**Figure 5**). The skull was cut along the coinciding point, and the distance between the puncture point and the puncture target was measured as the puncture depth (**Figure 6**).

#### 2.4.2 DSA machine panel operation

**Figure 7:**
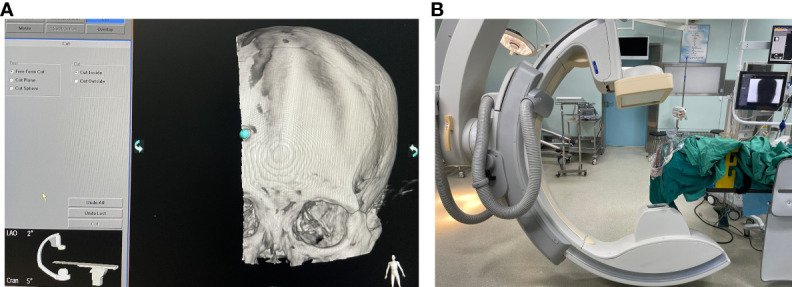
**(A)** Setting the puncture angle. **(B)** Xper CT rotation positioning.

The 3D APC function was used in the Xper APC automatic position control module to automatically position the rack movement to the angle displayed by the 3D reference image (**Figure 7**) (9).

#### 2.4.3 Surgical procedure

**Figure 8:**
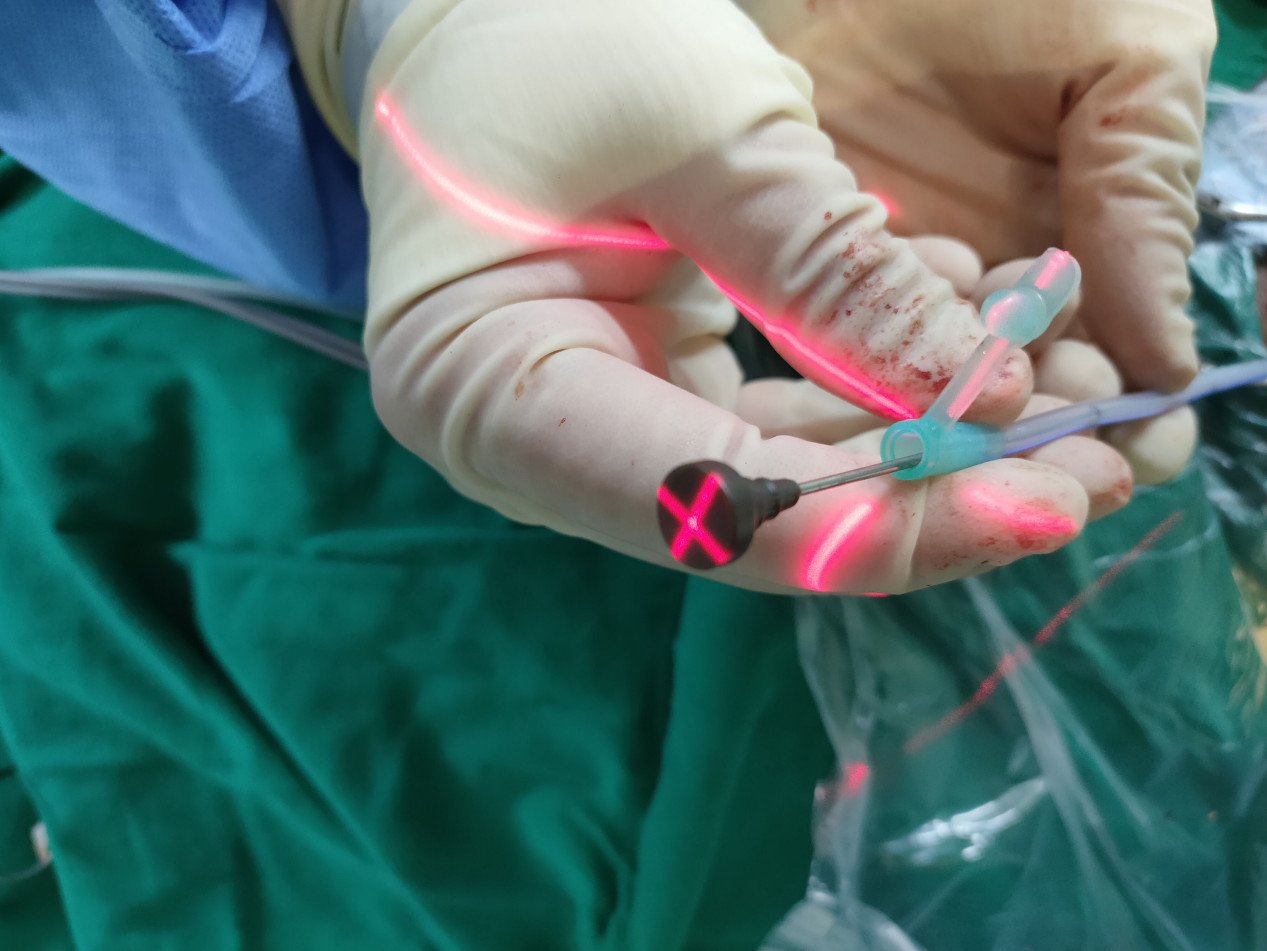
Puncture process.

A surgical incision was planned on the forehead scalp at the metal marker, with a length of approximately 2 cm. We stuck the bottom of the laser emitter on the FD plate, making the laser emitted from it perpendicular to the FD plate, then moved the laser emitter, and focused the cross-laser on the puncture point. Consequently, the direction of laser emission passed through the puncture point and puncture target simultaneously, which was determined as the puncture direction, and the puncture needle was held at the puncture point to conduct the puncture. During puncture, the laser focus was continuously placed at the center of the needle tail. At this time, the puncture of the trocar was continuously directed toward the center of the hematoma. Puncture depth was defined as the distance from the puncture point to the puncture target measured before surgery (**Figure 8**). Dark red blood flowed out through the drainage tube after the tube core was pulled out.

#### 2.4.4 Rechecking the position of the drainage tube

**Figure 9:**
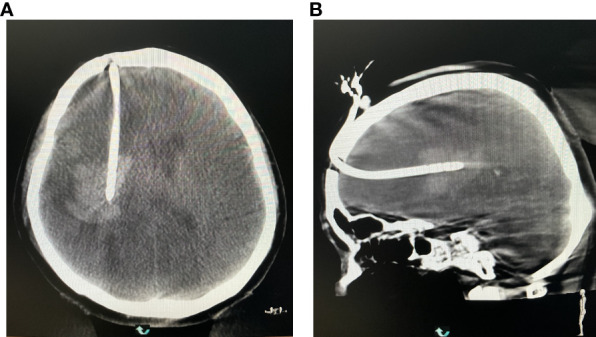
**(A)** Reexamining the puncture results in the coronal position. **(B)** Rechecking the puncture results at the axial position.

The DSA machine was used for CT scanning, collecting data, and transmitting the information to a computer. It was ensured that the drainage tube was located at the center of the hematoma (**Figure 9**).

### 2.5 Postoperative treatment

Both groups underwent the same postoperative treatments. A drainage tube was placed approximately 10 cm above the auricle for continuous drainage. No intracranial hemorrhage was found after 24 h on brain CT examination. Subsequently, 30,000 units of urokinase were completely dissolved in 5 ml of 0.9% sodium chloride and injected into the hematoma cavity. The drainage tube was closed for 2 h and then reopened. The urokinase solution was injected twice daily for 3 consecutive days. During the observation period, if the patient’s vital signs and consciousness deteriorated, drainage was performed promptly. On postoperative days 3, 5, and 7, brain CT was re-examined, and the change in the hematoma volume was observed and measured. The drainage tube was removed within 7–10 days according to drainage of the hematoma.

### 2.6 Efficacy evaluation

#### 2.6.1 Hematoma clearance rate

Hematoma clearance rate (10) = (preoperative hematoma volumeresidual — hematoma volume) / preoperative hematoma volume × 100%. Hematoma clearance rates at 3, 5, and 7 days after surgery were statistically analyzed.

#### 2.6.2 Clinical efficacy

The ADL grading (11) method was used to evaluate the clinical efficacy of the treatment, and the prognosis at 1, 3, and 6 months after surgery was calculated.

### 2.7 Statistical analysis

SPSS 21.0 software was used for statistical analysis. The *t*-test and rank sum test were used for data analysis, and the difference was considered statistically significant at *p* < 0.05.

## 3 Results

### 3.1 Comparison of hematoma clearance rate between the two groups

The hematoma clearance rates of the test group at 3, 5, and 7 days after surgery were significantly higher (*p* < 0.05) than those of the control group (**Table 1**). This result shows that the hematoma and pressure on the brain were significantly reduced at an early stage in the experimental group, with the restoration of the local blood supply to the brain tissue.

**Table 1:** Comparison of the hematoma clearance rates between the two groups.

Group	Number of cases	Hematoma clearance rate (%)		
		Postoperative 3 days	Postoperative 5 days	Postoperative 7 days
Control group	59	33.29 ± 6.98	47.90 ± 11.18	74.07 ± 7.31
Test group	59	36.24 ± 7.72	52.35 ± 10.26	77.37 ± 6.44
*Z*-value		2.18	2.25	2.60
*p*-value		0.031	0.026	0.011

### 3.2 Comparison of ADL grading evaluation results between the two groups at 1, 3, and 6 months postoperatively

One month postoperatively, the ADL grading of the patients in the test group was significantly better than that in the control group (*p* < 0.05), and the recovery of patients in the test group was also better than that in the control group.

However, at 3 and 6 months postoperatively, there was no significant difference in the ADL grading between the two groups (*p* > 0.05) **Table 2**), although there were more patients in the test group who recovered well (Grade I to Grade III) than those in the control group.

**Table 2:** Comparison of ADL grading between the two groups.

Group	Postoperative 1 month					Postoperative 3 months					Postoperative 6 months				
	I	II	III	IV	V	I	II	III	IV	V	I	II	III	IV	V
Control group	1	15	18	19	6	3	19	15	14	8	4	22	11	13	9
Test group	2	22	20	11	4	4	23	19	8	5	6	25	17	4	7
*Z* value	−1.965					−1.454					−1.475				
*p*-value	<0.05					>0.05					>0.05				

ADL, daily living ability.

## 4 Discussion

Hematoma puncture is an essential and effective surgical method for treating cerebral hemorrhage (12, 13). It has the advantages of a short operation time, simple operation, and minimal trauma. However, there are inevitable differences in the experience level of operators, resulting in variations in the puncture position, angle, and depth, as well as damage to the brain tissue and blood vessels by repeated punctures. At present, the commonly used penetration navigation methods include optical navigation, mechanical navigation, and electromagnetic navigation, but they all have some limitations (14). We used a 3D laser combined with C-arm CT to provide a convenient and accurate navigation method.

C-arm CT is a medical imaging technology that combines the advantages of two advanced technologies: the rotation of the DSA flat panel detector and computer reconstruction (15). Compared to CT-guided puncture, C-arm CT has certain advantages in the puncture of cerebral hemorrhage. Firstly, during the process of CT-guided puncture, the operator can only puncture according to the CT image through experience, which is subjective and can easily lead to repeated punctures and damage to the normal brain tissue and blood vessels.

C-arm CT integrates the effective functions of CT and DSA, plans the puncture path at multiple levels and angles, obtains the puncture information in real time during the puncture process, and observes the positional relationship between the puncture needle and hematoma at any time, providing real-time guidance during the puncture process (16). It not only reduces the puncture time but also ensures the accuracy and effectiveness of the puncture. Secondly, compared to spiral CT, the radiation dose in C-arm CT is reduced by approximately 60%–80% (17), thus reducing radiation damage to the target organs. Thirdly, patients need to be transferred to the traditional CT machine for routine postoperative CT re-examination. C-arm CT can be used to assess the intracranial condition of the patients immediately after surgery, without requiring transfer, and allows timely observation of surgical complications such as intracerebral rebleeding, which not only saves time but also allows faster treatment of complications.

Semiconductor lasers have the advantages of good monochromaticity, directivity, and brightness. Using the characteristics of high brightness and strong directivity of the laser, we fixed the semiconductor laser emitter to the flat panel detector of the DSA machine, making the laser beam perpendicular to the flat panel detector. Therefore, the laser beam could be parallel to the x-ray emitted by the flat panel detector, thus visualizing the x-ray that could not be observed. When the laser beam irradiates the puncture point on the head, it forms a bright cross-shaped stereo effect projection light that guides the puncture needle direction. During the operation, the laser direction, puncture needle, and target should be kept in the same line to achieve real-time guidance. Under laser guidance, the operator can accurately place the drainage tube at a predetermined position, which solves the shortcomings of minimally invasive puncture and drainage.

We used 3D laser navigation combined with C-arm CT puncture and drainage for early fibrinolysis treatment of hypertensive ICH. The hematoma clearance rates at 3, 5, and 7 days after surgery were significantly higher than those in the control group, indicating that this technology has a positive effect on reducing early hematoma and brain compression. There was also a statistically significant difference in the ADL grading between the test and control groups 1 month postoperatively. Although there was no statistically significant difference in ADL grading between the test group and control group at 3 and 6 months after surgery, there were more patients in the test group who recovered well (grades I to III) than in the control group. Therefore, compared to conventional CT guidance, 3D laser navigation combined with C-arm CT for intracerebral hemorrhage puncture is more time-saving, safer, more effective, and less traumatic, and results in faster recovery, a shorter hospital stay, and fewer complications, which is conducive to the development of minimally invasive technology and has considerable clinical value. In future studies, more clinical data will be collected and analyzed to further improve the reliability of the study.

## Data availability statement

The original contributions presented in the study are included in the article/supplementary material. Further inquiries can be directed to the corresponding authors.

## Ethics statement

The studies involving human participants were reviewed and approved by Ethics Committee of Binzhou Medical University Hospital. The patients/participants provided their written informed consent to participate in this study. Written informed consent was obtained from the individual(s) for the publication of any potentially identifiable images or data included in this article.

## Author contributions

HZ drafted the manuscript and was responsible for the revision and data processing of the article. TZ and SW completed the operation. ML and YG performed the data collection and data analysis. RJ and ZL participated in the design of this study and helped to check the manuscript. All authors contributed to the article and approved the submitted version.
